# Deuterium Metabolic Imaging of the Human Abdomen at Clinical Field Strength

**DOI:** 10.1097/RLI.0000000000001170

**Published:** 2025-03-17

**Authors:** Pascal Wodtke, Mary A. McLean, Ines Horvat-Menih, Jonathan R. Birchall, Maria J. Zamora-Morales, Ashley Grimmer, Elizabeth Latimer, Marta Wylot, Rolf F. Schulte, Ferdia A. Gallagher

**Affiliations:** Department of Radiology, University of Cambridge, Cambridge, UK (P.W., M.A.M., I.H.-M., J.R.B., M.J.Z.-M., A.G., E.L., M.W., F.A.G.); University of Cambridge, Cancer Research UK Cambridge Institute, Cambridge, UK (M.A.M., F.A.G.); and GE HealthCare, Munich, Germany (R.F.S.)

**Keywords:** deuterium, DMI, spectroscopy, ^2^H-glucose, metabolic imaging, molecular imaging, abdomen, liver, kidney

## Abstract

**Objectives:**

The aim of the study was to translate abdominal deuterium metabolic imaging (DMI) to clinical field strength by optimizing the radiofrequency coil setup, the administered dose of deuterium (^2^H)-labeled glucose, and the data processing pipeline for quantitative characterization of DMI signals over time. This was assessed in the kidney and liver to establish a basis for routine clinical studies in the future.

**Materials and Methods:**

5 healthy volunteers were recruited and imaged on 2 or 3 separate occasions, with varying doses of ^2^H-glucose: 0.75 g/kg (high dose), 0.50 g/kg (medium dose), and 0.25 g/kg (low dose), resulting in a total of 13 DMI scan sessions. DMI was performed at 3 T using a flexible 20 × 30 cm^2 2^H-tuned transmit-receive surface coil. For quantitative comparisons across scans, the ^2^H-glucose signal was normalized against the sum of ^2^H-glucose and ^2^H-water (GGW ratio). To quantify the time course of GGW, 3 novel metrics of metabolism were defined and compared between doses and organs: the maximum value across the time course (GGW_max_), the sum over the whole time course (GGW_AUC_), and the average signal across a defined plateau (GGW_mean plateau_). The ^2^H-lipid signal overlaps with ^2^H-lactate; hence, the 2 signals were measured as the combined ^2^H-lipid+lactate signal.

**Results:**

The careful positioning of a dedicated surface coil minimized unwanted gastric signals while maintaining excellent hepatic and renal measurements. The time courses derived from the liver and kidney were reproducible and comparable across different doses, showing the potential for dose reduction. The signal from the liver plateaued at approximately 30 minutes, and that from the kidney at approximately 40 minutes. The liver exhibited higher quantitative values for ^2^H-glucose uptake compared to the kidney, a trend consistent across all 3 quantitative metrics and doses, for example, for the highest dose: GGW_AUC liver_ = 31 ± 3; GGW_AUC kidney_ = 27 ± 3; *P* = 0.05. A trend toward lower quantitative measurements with decreasing dose was observed: this was significant between the high and the low dose for all 3 parameters and between the medium and low dose for GGW_mean plateau_ and GGW_AUC_, but was not significant between the high and the medium dose for any of the 3 parameters. The hepatic ^2^H-lipid+lactate signal increased over 70–90 minutes in 12/13 cases (mean: 39 ± 24%), while the renal lipid+lactate signal increased in only 8/13 cases (mean: 5 ± 17%). The hepatic ^2^H-water signal increased in all 13 cases (mean: 18 ± 10%), and the renal ^2^H-water signal increased in only 10/13 cases (mean: 10 ± 13%).

**Conclusions:**

DMI of the human abdomen is feasible using a clinical magnetic resonance imaging system and the signal changes measured in the kidney and liver can serve as a reference for future clinical studies. The ^2^H-glucose dose can be reduced from 0.75 to 0.50 g/kg to minimize gastric signal without substantially affecting the reliability of organ quantification. The increase in ^2^H-lipid+lactate or ^2^H-water signal over time could serve as direct and indirect measures of metabolism, respectively.

Imaging metabolic processes is crucial for understanding both normal physiology and disease, with alternations in glucose metabolism playing a central role in conditions, such as cancer and diabetes. Therefore, monitoring real-time glucose metabolism noninvasively holds transformative potential as a research tool and for clinical practice, enabling detailed insights into cellular function and disease progression. An important example of altered glucose metabolism is aerobic glycolysis, or the Warburg effect, which is observed in many cancers^1^: increased glucose consumption and lactate production, even despite an abundance in oxygen, creates an acidic extracellular environment that supports tumor growth. These metabolic shifts can be detected using novel metabolic magnetic resonance imaging (MRI) methods to noninvasively classify aggressive tumor subtypes and monitor early treatment responses.^2–5^

One emerging non-invasive method to assess glycolytic metabolism in the brain and cerebral tumors is deuterium metabolic imaging (DMI).^6^ Unlike conventional proton (^1^H) MRI, deuterium (^2^H) is found at very low natural abundance (~0.01%), and therefore, deuterium spectra contain almost no background signal apart from the large peak arising from the naturally abundant and partially deuterated water signal (^2^H-water or HDO), with some smaller deuterated peaks arising from adipose tissue. DMI exploits this low background signal through the administration of exogenous ^2^H-labeled molecules such as ^2^H-glucose ([6,6′-^2^H_2_]glucose).^7–11^ This labeled glucose is non-toxic^12^ and can be administered orally, which facilitates the clinical use of the technique (Fig. 1A). Following absorption in the gastrointestinal tract, ^2^H-glucose metabolism can be imaged over time using deuterium magnetic resonance spectroscopic imaging (^2^H-MRSI).^6,9^

**FIGURE 1:**
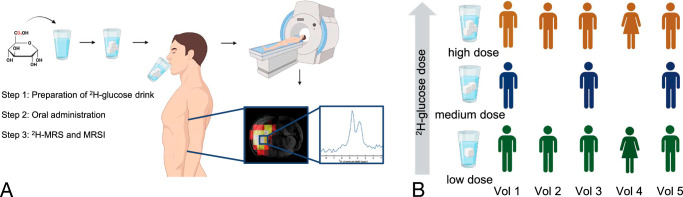
Study schema and experimental procedure. A, DMI as a technique. A solution containing ^2^H-glucose is prepared and orally administered, followed by spectroscopic imaging. B, n = 5 volunteers were imaged on 2 (n = 2) or 3 (n = 3) different days, totaling 13 imaging sessions. At each visit, the volunteers received 1 of 3 dose regimens of the ^2^H-glucose. DMI, deuterium metabolic imaging.

Despite having great potential for assessing glycolysis, DMI has faced several challenges in its translation to clinical field strength.^7,13^ While DMI has recently been translated to 3 T in the brain,^9,14^ DMI outside of the brain suffers from additional challenges which reduce spectral quality including: increased tissue inhomogeneity, motion, poor shimming, prominent gastric signal from the oral probe, and overlap between the naturally-abundant lipid peak and the tumor ^2^H-lactate signal. For these reasons, abdominal DMI has only been conducted at 7 T to date.^8,10^

In this study, we have addressed these challenges and conducted abdominal DMI in healthy volunteers at 3 T. The oral ^2^H-glucose dose has been varied to explore the optimal dose required for clinical imaging and to reduce both the cost and the gastric signal arising from the oral ingestion of the probe, without affecting the desired signal from the abdominal organs.^15^ We demonstrate the feasibility of quantitative abdominal DMI in the liver and kidney at clinical field strength and present a robust analytic framework for assessing ^2^H-glucose spectra for future clinical use, including novel metrics of metabolism.

## MATERIALS AND METHODS

### Study Design

The study was approved by a local research ethics committee (23/YH/0127) and all 5 healthy volunteers recruited gave informed consent in writing: 4 male, 1 female, age = 27 ± 2; Table 1. To assess the effect of dose reduction, the volunteers underwent up to 3 DMI studies on different days, receiving varying doses of ^2^H-glucose (Cambridge Isotope Laboratories Inc., Tewksbury, MA), dissolved in water (B. Braun, Melsungen, Germany), as shown in Table 1. The 3 dose regimens chosen for this study include the current standard dose given to volunteers and patients in previously published studies^9,11^ (“high”), as well as two-thirds (“medium”) and one-third (“low”) of the standard dose. The “high” dose corresponds to 0.75 g/kg, with a maximum administration of 60 g. The medium and low dose corresponds to 0.50 g/kg and 0.25 g/kg, with a maximum of 40 g and 20 g, respectively. All 5 volunteers received the high and the low dose, while 3 volunteers also received the medium dose, totaling 13 DMI sessions. The volunteers were fasted for 6 hours prior to scanning. Blood pressure, heart rate, blood oxygen saturation, and blood glucose level were recorded before and immediately after each exam (Fig. S1, http://links.lww.com/RLI/B6).

**TABLE 1:** Demographics

		^2^H-Glucose, g	Dose, g/kg	Dose Label	Age	Sex	Height, cm	Weight, kg	BMI, kg/m^2^
**Vol 1**	Visit 1	60.00	0.69	High	28	M	192	87.0	23.6
	Visit 2	40.00	0.46	Medium				87.0	23.6
	Visit 3	20.00	0.23	Low				87.0	23.6
**Vol 2**	Visit 1	20.00	0.19	Low	29	M	189	108.0	30.2
	Visit 2	60.00	0.56	High	30			107.2	30.0
**Vol 3**	Visit 1	40.00	0.50	Medium	23	M	187	80.0	22.9
	Visit 2	60.00	0.75	High				80.0	22.9
	Visit 3	19.55	0.25	Low				78.2	22.4
**Vol 4**	Visit 1	40.88	0.75	High	27	F	164	54.5	20.3
	Visit 2	13.50	0.25	Low				54.0	20.1
**Vol 5**	Visit 1	33.50	0.50	Medium	27	M	184	67.0	19.8
	Visit 2	50.18	0.75	High				66.9	19.8
	Visit 3	17.00	0.25	Low				68.0	20.1

Details about the individual volunteers and the dose received at each visit.

### ^2^H-MR Spectroscopy and Imaging

The volunteers were positioned supine in the MRI (3 T Premier, GE Healthcare, Chicago, IL) head-first. A flexible 20 × 30 cm^2 2^H-transmit-receive surface coil (RAPID Biomedical, Rimpar, Germany) was wrapped around the right side of the abdomen and fixed via Velcro straps, with the center of the coil positioned between the liver and kidney. An axial *T*_1_-weighted volume (LAVA-flex) was acquired for co-registration using the built-in body coil. Subsequently, a series of Hamming-filtered density-weighted ^2^H-MRSIs were acquired (real matrix size = 10 × 10 × 10, 1678 interleaves, field of view (FOV) = 40 cm, flip angle (FA) = 60°, echo time (TE) = 0.7 ms, repetition time (TR) = 250 ms). These have previously been shown to minimize Gibbs ringing and have a narrow point spread function,^16,17^ and were acquired from 70 to 90 minutes, interleaved between with the collection of unlocalized ^2^H spectra (TE = 0.7 ms, TR = 600 ms, FA = 90°, 64 averages).

### Data Processing and Analysis

Spectra were zero-filled by a factor of 2 in all 3 spatial dimensions and fitted automatically using a customized version of OXSA-AMARES^18,19^ with an SNR threshold of 5. To aid display in Figure 5, spatial interpolation by a factor of 12.8 in-plane, and a factor of 2 in the z-dimension, was conducted. Voxels were manually assigned to the respective organs using an overlay of the ^2^H-signal summed over all metabolites and the axial anatomical LAVA-flex ^1^H localizer (FOV = 48 cm, FA = 12°, TEs = 1.1/2.2 ms, TR = 3.96 ms), as seen in Figure 3. For liver, kidney, and duodenum, the prior knowledge of the fitting routine included the 3 peaks expected in DMI studies in the abdomen^10^: ^2^H-water, ^2^H-glucose, and ^2^H-lipid+lactate (whose peaks overlap). When assessing the stomach, the prior knowledge was set to detect only a single peak, assumed to be ^2^H-glucose in the absence of metabolism. Peak amplitudes, as fitted by OXSA-AMARES, were used to calculate the GGW and LLW (equation 1 and 2) ratios for every organ and time point using MATLAB (MathWorks, Natick, MA). The GGW and LLW ratios represent the respective normalization of ^2^H-glucose and ^2^H-lipid+lactate (liplac) signals against the naturally abundant ^2^H-water peak to permit a comparison across individuals, as defined in equations 1 and 2:

**FIGURE 2:**
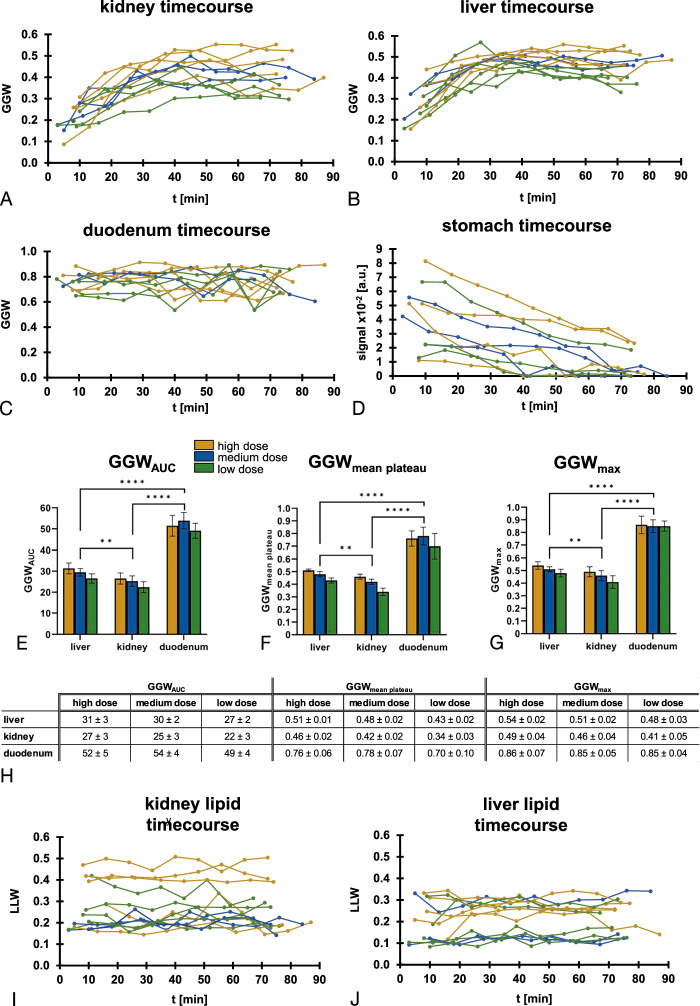
Dynamic unlocalized spectroscopy acquired from the healthy abdomen after oral administration of ^2^H-glucose. A–C, Spectral time courses demonstrating ^2^H-water and ^2^H-glucose peaks from a single volunteer (#3) after receiving the following ^2^H-glucose doses: (A) high, (B) medium, and (C) low. D, Time courses of GGW (see methods) for each volunteer at each visit. E, The 3 different quantitative measures we have introduced to evaluate ^2^H-glucose signal over time (see methods). F, The total ^2^H-glucose signal initially increases with respect to the first time point in all volunteers and starts to decline after ~50–60 minutes. G, The ^2^H-water grows moderately over time in all volunteers, indicating the production of water through metabolism. H, First (start) versus last (end) time point of the data shown in (G), to demonstrate that all volunteers exhibit a growth in the ^2^H-water signal, suggesting a consistent effect.

**FIGURE 3:**
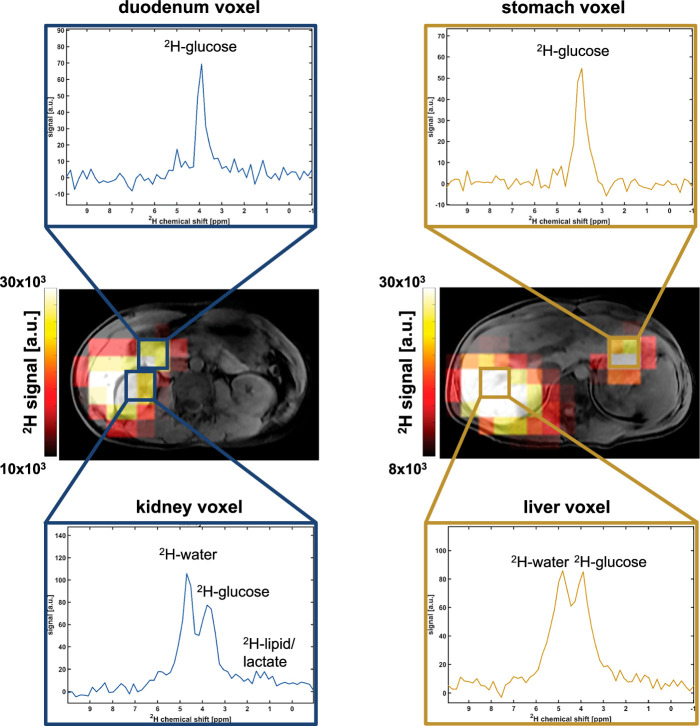
Representative images derived from spatially resolved spectroscopy. The summed ^2^H signal is shown as a color scale image with single-voxel spectra from each organ (volunteer 5, *t* = 33 minutes after receiving the high [gold] and the medium [blue] doses). Stomach and duodenal regions of interest are dominated by the ^2^H-glucose signal, whereas kidney and liver exhibit a prominent ^2^H-water peak as well as a small lipid peak in the kidney. The boxes in the images indicate noninterpolated voxel sizes.


$$\mathrm{GGW}\left(t\right)=\frac{\text{glucose}\left(t\right)}{\text{glucose}\left(t\right)+\text{water}\left(t\right)}\;$$ equation 1

$$\mathrm{LLW}\left(t\right)=\frac{\text{liplac}\left(t\right)}{\text{liplac}\left(t\right)+\text{water}\left(t\right)}$$ equation 2

with glucose(t), liplac(t), and water(t) representing the ^2^H-glucose, ^2^H-lipid+lactate, and ^2^H-water signals at the respective time points. A ratiometric approach, as opposed to raw signals, was chosen to compensate for the spatial inhomogeneity of the surface coil and hence be comparable across the field-of-view. The GGW provides a normalized metric of ^2^H-glucose uptake, which is comparable across voxels and participants as a potentially useful clinical measurement. LLW is a similar comparative metric, which at baseline is dominated by the lipid signal, and any increase over time corresponds to lactate formation in the organ of interest. For quantitative analysis of the time courses, GGW_max_, GGW_AUC_, and GGW_mean plateau_ were calculated. GGW_max_ was determined by taking the maximum GGW value in each organ time course. For GGW_AUC_, the GGW value of each time point *t_n_* was multiplied by the time difference *t_n_-t_n-1_* between the previous time point in minutes (equation 3). The separate AUC (*t*) values were then summed until *t* = 74 min, which was a common time point across volunteers and exams, to make the GGW_AUC_ comparable across volunteers and across scans:


$${\mathrm{GGW}}_{\mathrm{AUC}}\left(x\right)=\sum_{n=1}^{x}\mathrm{GGW}\left(t_{n}\right)˙\left(t_{n}-t_{n-1}\right);\text{with}\;t_{x}=74\;\mathrm{minutes}$$ equation 3

GGW_mean plateau_ was determined by taking the mean GGW of all time points after signal plateauing. The plateau time was determined to be *t* = 30 minutes for liver and *t* = 40 minutes for kidney, broadly aligning with previously published studies.^10^ For the unlocalized spectroscopy, the GGW_mean plateau_ was replaced by GGW_mean_ over the whole time course, because the detection of signal build-up and subsequent plateauing were not possible for all scans, depending on the contribution of the stomach to the overall detected signal. Analysis of variance (ANOVA) was performed in GraphPad Prism (GraphPad Software, Boston, MA), followed by Tukey's honestly significant difference (HSD) test as post hoc analysis. To test for significance of the increase in localized ^2^H-lipid+lactate and ^2^H-water signals, a 1-sample Wilcoxon signed-rank test was conducted and the sum of signed ranks displayed in brackets (W).

## RESULTS

### Unlocalized Spectroscopy

Unlocalized spectra showed good SNR (>5) for all 3 dose regimens, with the ^2^H-water (4.7 ppm) and ^2^H-glucose (3.7 ppm) peaks being clearly resolved at clinical field strength (Figs. 2A–C). Interestingly, the GGW ratio derived from these unlocalized spectra showed ^2^H-glucose build-up steadily over time in some volunteers, while appearing earlier in others (Fig. 2D). This is because, depending on the coil positioning, the stomach and duodenum were detectable in some cases, resulting in early and high ^2^H-glucose signal in some cases. The low dose regimen exhibited a lower GGW across all metrics (Fig. 2E; GGW_max_ = 0.44 ± 0.03; GGW_mean_ = 0.35 ± 0.05; GGW_AUC_ = 24 ± 5; n = 4) compared to the medium (GGW_max_ = 0.55 ± 0.07, *P* = 0.09; GGW_mean_ = 0.48 ± 0.05, *P* = 0.02; GGW_AUC_ = 29 ± 3; *P* = 0.51, n = 3) and the high doses (GGW_max_ = 0.57 ± 0.07, *P* = 0.02; GGW_mean_ = 0.47 ± 0.05, *P* = 0.01; GGW_AUC_ = 34 ± 6, *P* = 0.06; n = 5). Statistically significant results are indicated in Fig. 2E. Notably, there was no significant difference in the quantitative values between the medium and the high dose across all the metrics. The dynamics of the fitted ^2^H-glucose signal amplitude showed a signal build-up over 60 minutes in most cases (Fig. 2F). An increase in the ^2^H-water signal over time could be observed in all cases, ranging from 10% to 56% when comparing the last time point with the first in each case (Figs. 2G–H and S2, http://links.lww.com/RLI/B6).

**FIGURE 4:**
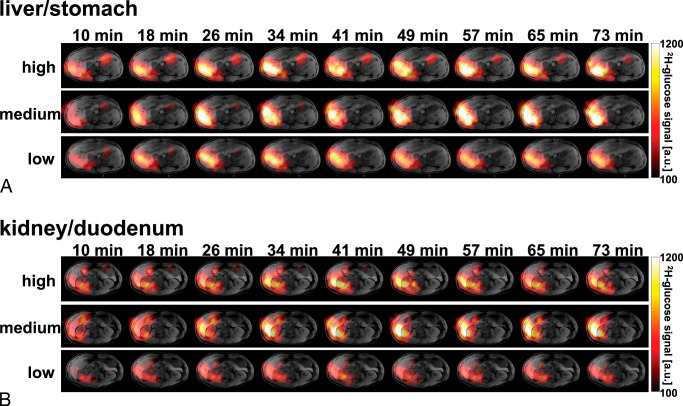
Quantitative evaluation of imaging data. A–C, GGW time courses for ^2^H-glucose signal in the kidney (A), liver (B), and duodenum (C). D, ^2^H-glucose signal in the stomach over time shows a decrease likely to correspond to gastric emptying. E–G, Quantitative measures of GGW_AUC_ (E), GGW_mean plateau_ (F), and GGW_max_ (G), exhibit dose dependence in the liver and kidney, whereas they remain elevated in the duodenum regardless of dose. H, Summarized quantitative values displayed in (E–G). I and J, ^2^H-lipid signal, as evaluated by the LLW parameter (see methods) over time. The kidney (I) generally shows a higher LLW than the liver (J), indicating a higher lipid content inside the imaged voxels, which may arise from the surrounding visceral fat.

### Imaging

The ^2^H signal was anatomically localized to the liver and kidney on MRSI in all cases (n = 13; Fig. 3) and in the stomach and duodenum in most cases (n = 10 and 11, respectively). Only ^2^H-glucose could be detected in the stomach, while the duodenum exhibited a small but detectable ^2^H-water peak. The kidney exhibited a small lipid peak, which was probably secondary to partial volume effects from the surrounding visceral fat.

The time courses for the kidney (Fig. 4A) and the liver (Fig. 4B) showed a build-up in the GGW ratio, with the liver plateauing earlier at ~30 minutes, compared to the kidney at ~40 minutes. GGW signal in the duodenum remained high throughout the whole time course (Fig. 4C). The raw or nonnormalized ^2^H-glucose signal was assessed in the stomach due to the low ^2^H-water signal, which showed a decrease over time in all cases as gastric emptying occurred (Fig. 4D).

**FIGURE 5:**
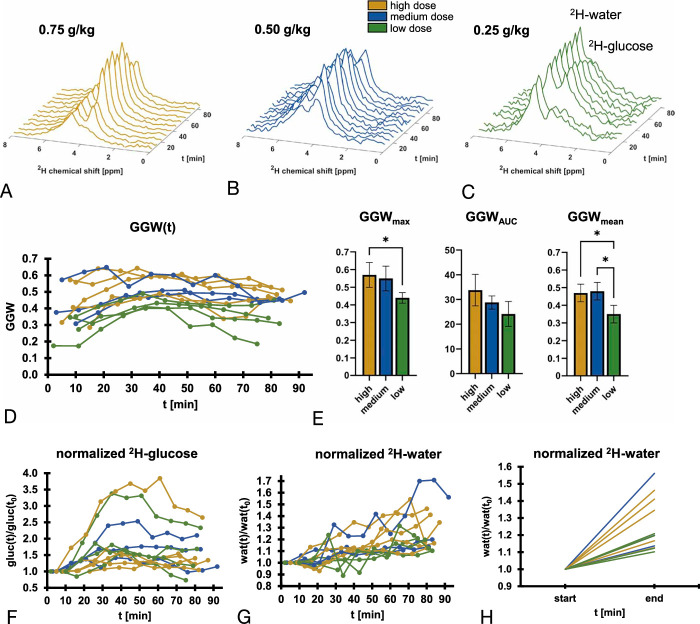
Imaging time courses for ^2^H-glucose for all 3 dose regimens administered to volunteer 5. A, A slice containing both the liver and stomach showing declining ^2^H-glucose signal in the stomach and increasing signal in the liver. B, A slice containing both the kidney and the duodenum: the kidney shows a build-up of ^2^H-glucose, whereas the duodenum signal shows little variation in signal intensity over time. Time courses of the ^2^H-water signal, as well as the summed ^2^H-signal can be viewed in the supplementary information (Fig. S4, S5, http://links.lww.com/RLI/B6).

### Comparison Between the Liver and Kidney, and Between Doses

The liver exhibited a significantly higher ^2^H-glucose uptake than the kidney across all 3 dose regimens and quantitative metrics (Figs. 4E–H, *P* values for GGW_max_, GGW_mean plateau_, GGW_AUC_: 0.01, <0.01, <0.01, respectively). Furthermore, all 3 values increased with dose in both the liver and kidney. Notably, this was significant between the high and the low dose for all 3 parameters and between the medium and low dose for GGW_mean plateau_ and GGW_AUC_, but was not significant between the high and the medium dose for any of the 3 parameters. Relevant *P* values are shown in Figure S3, http://links.lww.com/RLI/B6. Example imaging time courses from volunteer 5 for all 3 doses are displayed in Figure 5.

### ^2^H-Lipid Content and ^2^H-Lactate Formation

The ^2^H-lactate signal appears at a similar chemical shift as the ^2^H-lipid signal (both at ~1.3 ppm)^20^ as lipid is particularly abundant in the abdomen,^21,22^ and this presents a challenge for the accurate quantification of ^2^H-lactate. Hence, it is more important to characterize the naturally abundant lipid signal on abdominal DMI, compared to the brain where the majority of previous DMI research has been undertaken.

A small ^2^H-lipid peak was identified in voxels overlying the kidney, and to a lesser extent the liver, which was likely due to partial volume effects from the visceral fat surrounding these organs (Fig. 3). The LLW ratio was calculated and the respective time courses for both organs and are displayed in Figures 4I and J. The mean lipid content (across all volunteers and time points) was higher in the kidney (0.27 ± 0.10) compared to the liver (0.21 ± 0.08) but this trend was not statistically significant (*P* = 0.10).

**FIGURE 6:**
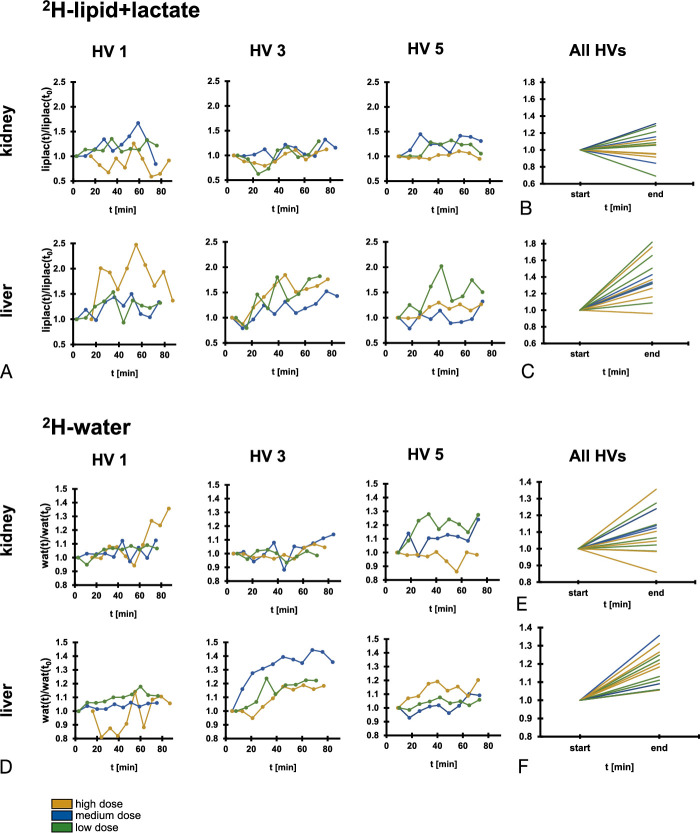
^2^H-water and ^2^H-lipid+lactate signal derived from individual volunteers over time, extracted from the imaging data. A, ^2^H-lipid+lactate signal from the 3 volunteers that underwent all 3 imaging sessions. Unlike the kidney, the liver showed a trend toward an increasing signal over time, up to ×2.5 the initial value. B and C, Comparison of the first and last time points from all the volunteers reveals a trend toward increasing ^2^H-lipid+lactate signal in the liver (C), whereas the kidney (B) shows no trend across volunteers. The liver data suggests the hepatic formation of ^2^H-lactate from ^2^H-glucose in the time course of the experiment. D, Comparative ^2^H-water signal of the same 3 volunteers. The liver shows a clear and consistent increase in ^2^H-water signal over time. E and F, Comparison of the first and last time points from all the volunteers reveals a trend toward increasing ^2^H-water signal in both kidney (E) and liver (F) over time. In the case of the liver, the ^2^H-water signal increased in all volunteers.

Lactate plays an important role in many pathological processes, and it is therefore key to quantify the background ^2^H-lactate signal derived from normal organs after oral administration of ^2^H-glucose as a reference. Figure 6A shows the normalized ^2^H-lipid+lactate signal for the 3 volunteers that were administered all 3 doses. A direct comparison of the last time point compared to the first for all volunteers can be seen in Figures 6B and C. In the liver, the ^2^H-lipid+lactate signal increased in 12 of the 13 examinations, with an average significant increase of 39 ± 24% (median = 34%, W = 89, *P* < 0.01) showing that the healthy liver exhibits detectable ^2^H-lactate 70–90 minutes after oral ^2^H-glucose administration. In contrast, the ^2^H-lipid+lactate signal increased in only 8 of the 13 examinations in the kidney, with an average increase of only 5 ± 17%, which was not significant (median = 7%, W = 33, *P* = 0.27), suggesting very little lactate formation in this timescale.

### ^2^H-Water Signal Increase as an Indirect Measure of Metabolism

The unlocalized spectroscopy showed an increase in ^2^H-water signal over time (Figs. 2G, H), which has been reported in previous preclinical and clinical studies.^9,10,23^ Spectroscopic imaging was subsequently used to localize this signal increase; Figure 6D shows the normalized ^2^H-water signal for the 3 volunteers that were administered all 3 doses. A direct comparison of the last time point compared to the first for all volunteers can be seen in Figures 6E and F. In the kidney, the ^2^H-water signal increased in 10 of the 13 examinations over time, with an average significant increase of 10 ± 13% (median = 10%, W = 67, *P* = 0.02). In the liver, the ^2^H-water signal increased in all 13 examinations, with an average significant increase of 18 ± 10% (median = 18%, W = 91, *P* < 0.01). Hence, the liver is likely driving the ^2^H-water signal increase in the unlocalized spectroscopy data. Indeed, the 3 examinations with the largest ^2^H-water signal increases in the unlocalized spectra were the same examinations that exhibited the largest ^2^H-water signal increase in the liver imaging data. This observation further confirms the conclusion that the liver is dominating the ^2^H-water signal increase in the unlocalized spectra.

## DISCUSSION

Deuterium metabolic imaging is an emerging method for probing tissue metabolism with potential applications in a wide range of diseases given the importance of glucose utilization in both health and disease. Here, we translate the technique to the abdomen at clinical field strength, showing its feasibility for assessing hepatic and renal metabolism. We demonstrate that the dose of oral ^2^H-glucose, and consequently the cost of a clinical DMI scan, can be significantly reduced to 0.5 g/kg (medium dose) without compromising spectral quality or quantitative accuracy, and present metrics to quantitatively evaluate its uptake and metabolism in tissues.

The signal from ^2^H-glucose was evaluated up to 90 minutes after oral administration and could be used to evaluate accumulation and absorption in the stomach and duodenum. Importantly, the use of a surface coil in combination with its positioning around the right side of the abdomen was used to minimize unwanted gastric signal when imaging the liver and right kidney. The liver generally exhibited a higher ^2^H-glucose signal compared to the kidney, rapid plateauing of the signal, more reproducible time curves, and a lower lipid signal, therefore making it a promising organ to be assessed using DMI.

The increase in the ^2^H-lipid+lactate signal observed in the liver is likely to be attributed to lactate formation during glycolysis as lipogenesis occurs on a slower timescale.^24^ The lipid signal demonstrates a low SNR and has a broader linewidth compared to the ^2^H-water and ^2^H-glucose signals and may be overestimated while fitting with the automated approach here, which calculates a peak area.

The increase in ^2^H-water signal over time is very likely to arise from water derived from glucose metabolism.^25,26^ The metabolic pathways that most likely contribute to this ^2^H-water signal increase are glycolysis^27,28^ and oxidative phosphorylation with deuterons being released as HDO.^7,9^ The liver showed a higher ^2^H-glucose uptake than the kidney, as well as the formation of a small ^2^H-lipid+lactate signal, in keeping with increased hepatic metabolism. The statistical analysis conducted to examine the increase in ^2^H-water and ^2^H-lipid+lactate signals assumed independence of the individual measurements. However, some of these measurements were taken from the same volunteers on different days and after different doses, so they may exhibit some degree of correlation. When the measurements were disaggregated by dose, the increases in ^2^H-lipid+lactate and ^2^H-water within the liver remain higher than those in the kidney, which provides strong support that the signal change in the liver is at least partially arising from true hepatic metabolism in addition to any contribution from metabolites that are being washed in from other organs (Fig. S6, http://links.lww.com/RLI/B6). The latter is a confounder when analyzing DMI data as the long time course for acquisition allows for metabolites from other organs to be washed into the organ under assessment, so the measured signal may not be due to metabolism in the organ under investigation. Furthermore, due to limited spatial resolution, glucose uptake in the renal parenchyma cannot be distinguished from accumulation in the collecting system, although the latter should be reabsorbed in normal physiological conditions. The ^2^H-glucose signal therefore reflects both intracellular or extracellular glucose accumulation, and true metabolism can only be inferred through downstream products such as ^2^H-water and ^2^H-lactate.

Although the volunteers in this study were not selected based on BMI, most had a normal BMI (<24), except for 1 individual with a BMI of 30. An increase in adiposity is expected to amplify the lipid signal, which could potentially interfere with the detection of ^2^H-lactate. The doses of ^2^H-glucose that have been used in DMI range from 60 to 75 g and may be dependent on weight,^8–11,29^ with 75 g being the standard dose for a clinical glucose tolerance test.^30^ The blood glucose is transiently increased during these dose regimens, and we show that this could be avoided by lowering the dose to ~20 g, allowing steady state metabolism to be probed without a substantial loss in signal (Fig. S1, http://links.lww.com/RLI/B6). For abdominal applications, the medium dose (0.50 g/kg, maximum 40 g) is particularly advantageous as it minimizes the risk of ^2^H-signal contamination from gastric compartments while maximizing the signal detected. This reduces the cost of DMI, as well as the risk of unwanted gastric signal contaminating the quantification from other organs.

As an emerging technique, the best approaches for quantitative assessment and kinetic modeling of DMI are yet to be determined. The challenges are similar to other time-resolved metabolic imaging methods, such as those found in the analysis of positron emission tomography (PET)^31^ and hyperpolarized ^13^C-MRI data.^32,33^ However, DMI also presents some problems unique to the technique and we have proposed 3 novel quantitative biomarkers for assessment of this dynamic data here: GGW_AUC_, GGW_mean plateau_, and GGW_max_. The results show that the hepatic and renal measurements are influenced by the administered dose but do not scale with it, suggesting a rate limiting element in delivery or metabolism. It should be noted that the GGW metric, when observed over a time course, can also be non-linear, as it involves dividing an increasing signal (^2^H-glucose) by 2 signals that are also increasing (^2^H-glucose + ^2^H-water). It is interesting to note that despite the range of doses across different volunteers, the duodenal measurements are remarkably constant over time and between individuals.

One limitation of this study is the small cohort size; extending this study into larger cohorts and undertaking formal repeatability and reproducibility studies in a multisite setting will be important for future research in this field. Normalized plots have been referenced to the first time point of the same exam and this reference time point differed across examinations. It would be advantageous to reference the ^2^H-water and ^2^H-lipid signals against the naturally abundant measurements at baseline, but as the participants were repositioned after drinking in the upright position, the exact placement of the coil could change, potentially resulting in misregistration. Future studies could overcome this by maintaining the participant on the scanner with the coil in position and administering the ^2^H-glucose using a straw,^8^ allowing the natural abundance scan to be directly compared to the postadministration images.

Imaging of different anatomical sites would require adapted coil placement. For example, to image both kidneys, the coil can be placed horizontally beneath the patient in a supine position. The left lobe of the liver, spleen, pancreas, and gastrointestinal organs, however, pose greater challenges. The ^2^H-glucose dose could be reduced, or imaging could be delayed until the gastric signal has sufficiently decreased or depleted (see Fig. 4D). However, accurately distinguishing these organs and quantifying them independently of the gastric signal may not always be feasible. In some cases, quantifying gastric signal or emptying might even be of interest, as it represents a physiological process. When gastric signals need to be avoided entirely, alternative administration routes, such as intravenous administration, must be explored. This would bypass gastrointestinal uptake, could potentially reduce the required dose further, and may lead to more rapid substrate delivery. However, it may result in a transient supraphysiological dose of glucose which could alter tissue metabolism.

An alternative way of obtaining information about the metabolic utilization of ^2^H-glucose, while negating the requirement for a dedicated ^2^H-coil, is quantitative exchanged label turnover (QELT),^34^ where the reduction in ^1^H-signal from relevant metabolites after the administration of ^2^H-glucose serves as an indirect measure of metabolism. This approach has recently been translated to clinical field strength but has been limited to the brain.^35,36^ While QELT does not require specialized hardware and offers potentially higher spatial resolution, the technique produces negative contrast, which is less intuitive to interpret, and results in a loss of SNR when the desired effect occurs, which is therefore more difficult to detect. The signal drop in QELT is small, and fitting metabolites accurately due to the crowded ^1^H-background spectra can be challenging, whereas ^2^H-spectra are sparse. Additionally, QELT always requires a baseline scan, whereas a single DMI scan could provide a binary answer to the question of whether a significant portion of the administered ^2^H-glucose is being converted into ^2^H-lactate.

## CONCLUSIONS

In conclusion, these data demonstrate the feasibility of quantitative DMI in the kidney and liver at clinical field strength, without signal interference by the stomach. Furthermore, we show that the administered dose can be significantly decreased, reducing the costs of a DMI study and the dominant gastric signal, which are important considerations for future clinical use. Additionally, we introduced new methodology to assess glucose uptake and metabolism, which could be used to quantify tissue metabolism in disease settings.

## Supplementary Material

SUPPLEMENTARY MATERIAL
