# Supplementary material for: Deuterium Metabolic Imaging of the Human Abdomen at Clinical Field Strength
Source: Invest Radiol. 2025 Mar 17;61(1):1–9. doi: 10.1097/RLI.0000000000001170 (PMC12662132; doi:10.1097/RLI.0000000000001170)
Supplement: SUPPLEMENTARY MATERIAL [file rli-61-01-s001.docx]

# Deuterium metabolic imaging of the human abdomen at clinical field strength

# Supplementary Information


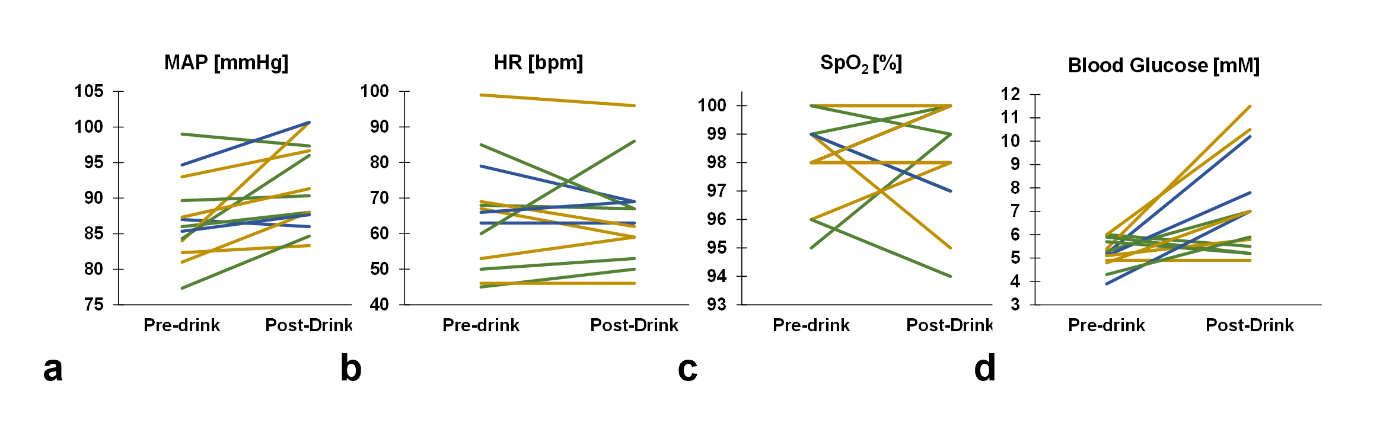


**Fig S1 | Physiological data acquired pre- and post-drink from the healthy volunteers.** MAP: mean arterial pressure; HR: heart rate; SpO_2_: oxygen saturation.


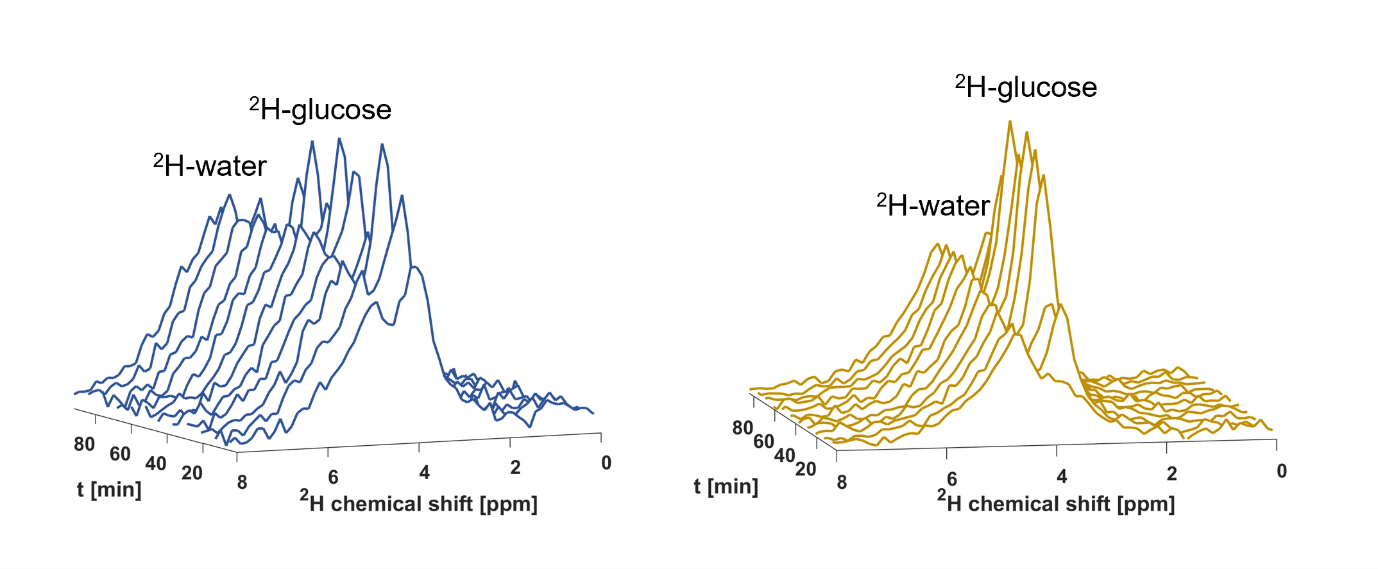


**Fig S2 | Representative spectra acquired from volunteer 3 after administration of medium (left) and high (right) dose, demonstrating a steady increase in the HDO signal over time.**


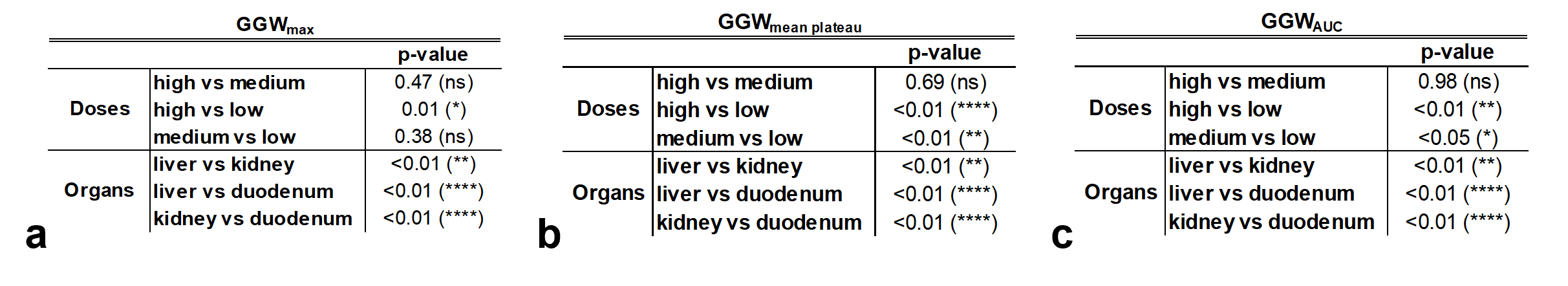


**Fig S3 | Statistical significance of MRSI data between doses and organs. (a) GGW_max_; (b) GGW_mean plateau_; (c) GGW_AUC_**.


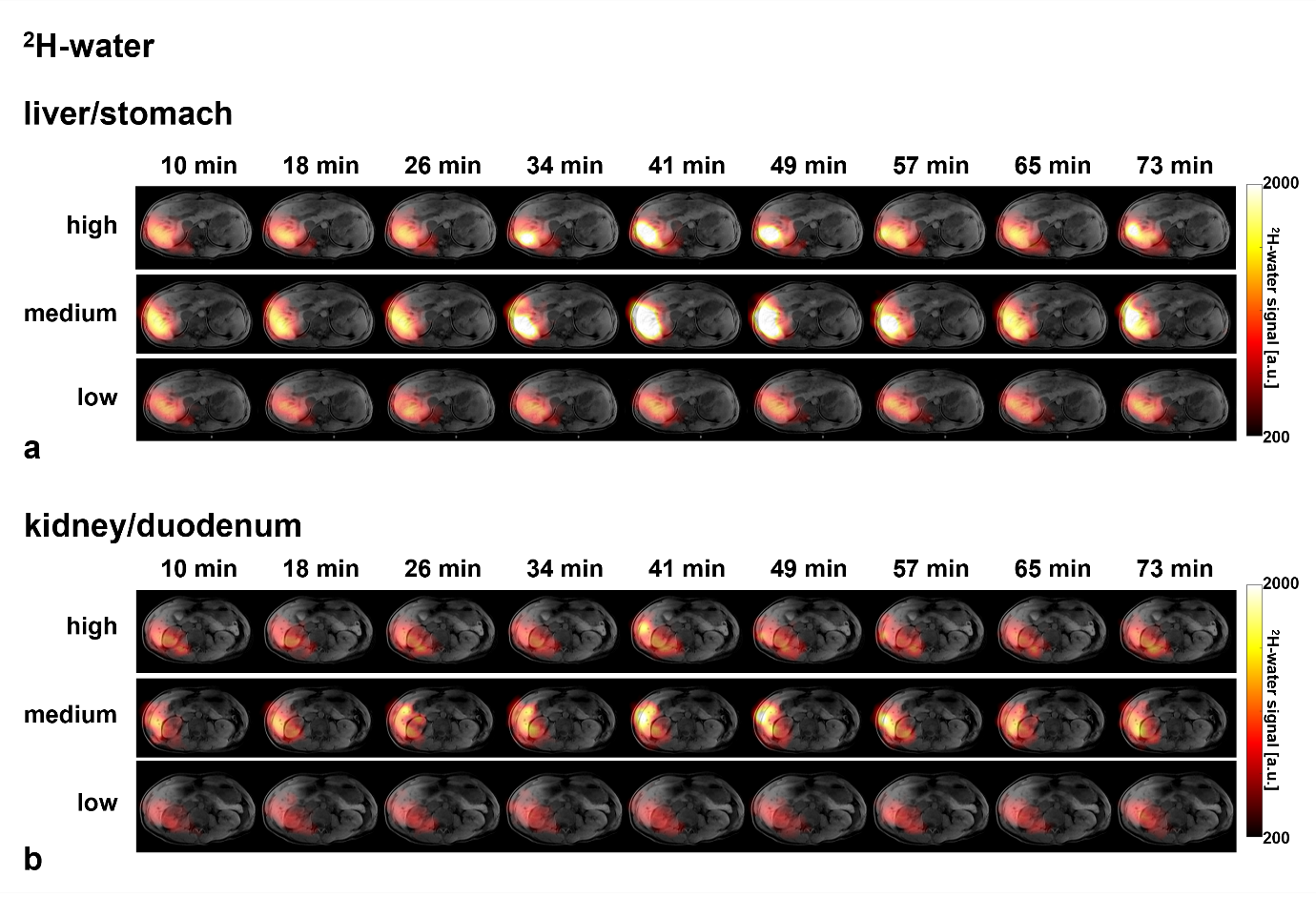


**Figure S4 | Imaging timecourses of the HDO signal for all three doses administered to volunteer 5.**


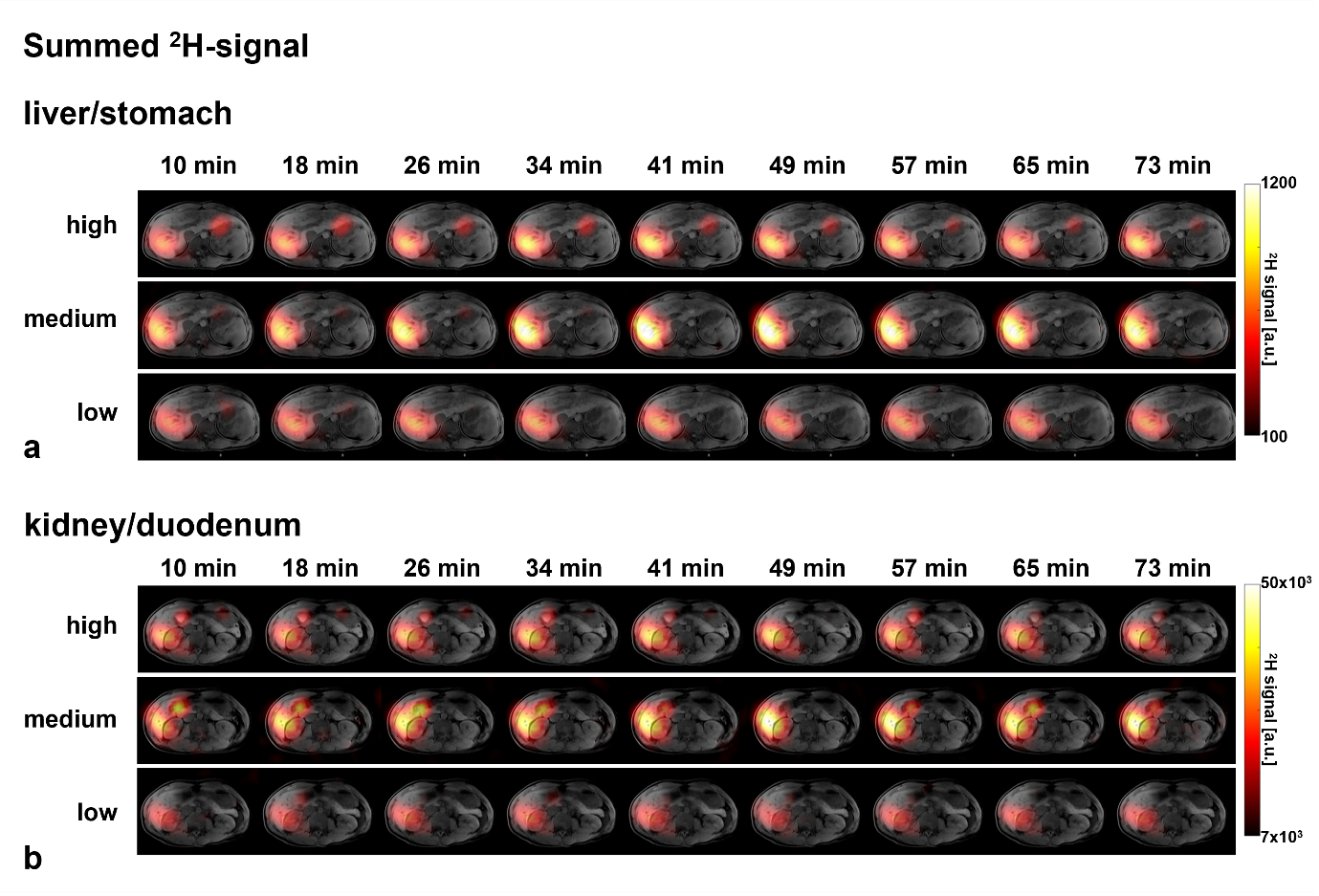


**Figure S5 | Imaging timecourses for the summed ^2^H-signal for all three doses administered to volunteer 5.**


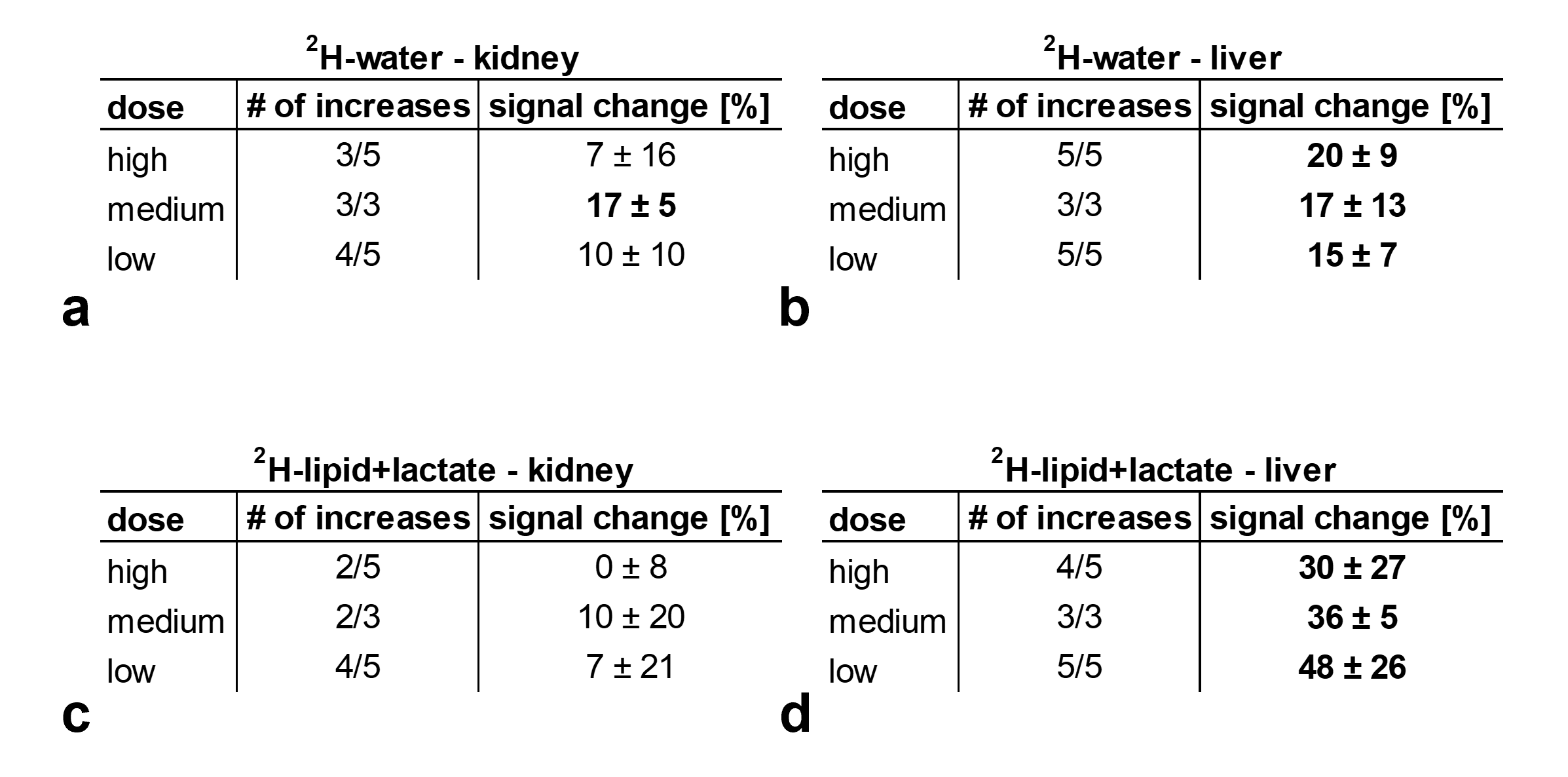


**Figure S6 | ^2^H-water (a, b) and ^2^H-lipid+lactate (c, d) signal changes in percent within 70-90 min separated by dose.** The tables display the number of cases where signal increased and the overall signal change in percent. **Bold** numbers indicate signal changes higher than their standard deviation.
